# Financial toxicity among patients with lung cancer: a systematic review and meta-analysis

**DOI:** 10.3389/fpubh.2026.1896259

**Published:** 2026-08-13

**Authors:** Min Jiang, Fangfang Wang, Yumeng Liu, Qi Hu, Linlin Zhao, Yu Xiong, Yuan Bian

**Affiliations:** 1 Department of Pharmacy, West China School of Public Health and West China Fourth Hospital, Sichuan University, Chengdu, China; 2 Department of Pharmacy, Emeishan People’s Hospital, Emeishan, China; 3 Department of Pharmacy, Daping Hospital, Army Medical University, Chongqing, China; 4 Department of Pharmacy, Hospital of Chengdu University of Traditional Chinese Medicine, Chengdu, China; 5 First Teaching Hospital of Tianjin University of Traditional Chinese Medicine, Tianjin, China; 6 Department of Pharmacy, Mianyang Central Hospital, School of Medicine, University of Electronic Science and Technology of China, Mianyang, China; 7 Sichuan Provincial Engineering Research Center of Nuclear Medical Equipment Translation and Application, Mianyang Central Hospital, Mianyang, China; 8 Department of Pharmacy, Personalized Drug Research and Therapy Key Laboratory of Sichuan Province, Sichuan Provincial People’s Hospital, School of Medicine, University of Electronic Science and Technology of China, Chengdu, China; 9 Department of Biotherapy,Cancer Center and State Key Laboratory of Biotherapy,West China Hospital, Sichuan University, Chengdu, China

**Keywords:** Comprehensive Score for Financial Toxicity, financial toxicity, lung cancer, meta-analysis, systematic review

## Abstract

**Background:**

Lung cancer imposes a substantial global health burden. Although advances in treatment have improved outcomes, increasingly prolonged and complex care may impose considerable financial pressure on patients and their families. We systematically synthesized evidence on financial toxicity (FT) and its associated factors among patients with lung cancer.

**Methods:**

PubMed, Embase, the Cochrane Library, and Scopus were systematically searched through November 2025 for observational studies evaluating FT in patients with lung cancer. Associated factors were synthesized qualitatively and mapped using a harvest plot. Random-effects models were used to pool study-specific FT proportions and mean COST-based scores. Exploratory subgroup analyses were conducted by country income level, disease stage/treatment setting, and COST-based FT threshold. Heterogeneity was assessed using Cochran’s Q and I² statistics. Prediction intervals and leave-one-out analyses were used to evaluate variability and robustness.

**Results:**

Eighteen studies were included. The pooled proportion of patients meeting study-specific FT definitions across eight studies was 0.55 (95% CI, 0.43–0.66), while the pooled mean COST-based score across ten studies was 22.91 (95% CI, 20.57–25.25). The pooled FT proportion was numerically lower in high-income than in upper-middle-income countries (0.47 vs 0.61; *p* = 0.171). FT proportions differed by disease stage/treatment setting (advanced/systemic treatment, 0.50; mixed/all-stage populations, 0.67; surgery-dominant populations, 0.42; *p* = 0.031). No difference was observed between studies using COST-based thresholds of 21–22 and 23–25 (*p* = 0.999). Lower income, inadequate insurance protection, employment disruption, limited savings, and greater out-of-pocket burden were recurring correlates of worse FT. Substantial heterogeneity persisted across analyses.

**Conclusion:**

FT represents an important but highly context-dependent burden among patients with lung cancer. Given the substantial heterogeneity and predominantly cross-sectional evidence, the pooled estimates should be interpreted as exploratory summaries rather than universally applicable benchmarks. Standardized longitudinal assessment and prospective evaluation of risk-stratified interventions are warranted.

## 1 Introduction

Lung cancer remains one of the most serious global public health challenges. According to Global Cancer Observatory (GLOBOCAN) 2022, there were approximately 2.48 million new lung cancer cases and 1.82 million deaths worldwide, making lung cancer both the most commonly diagnosed malignancy and the leading cause of cancer-related death globally (1, 2). Recent burden projections further suggest that, unless prevention, early detection, and treatment strategies improve substantially, the absolute number of lung cancer cases and deaths will continue to rise over the coming decades (2).

At the same time, the therapeutic landscape of lung cancer has changed markedly. Advances in surgery, radiotherapy, molecularly targeted therapy, and immune checkpoint inhibitors have improved outcomes for selected patient populations and expanded survivorship. However, these gains have also been accompanied by increasingly complex care pathways, prolonged treatment duration, repeated imaging and monitoring, and higher direct and indirect costs. As a result, attention has shifted beyond traditional endpoints such as survival and tumor response toward patient-centered outcomes, including quality of life, psychosocial well-being, and treatment affordability. Against a background of constrained public finances, a recent Organization for Economic Co-operation and Development (OECD) report emphasized the growing importance of improving health-system efficiency, controlling pharmaceutical expenditure, and reforming payment mechanisms. These pressures have increased attention to the sustainability and equity of health-care financing, including whether rising costs are transferred to patients and their families (3). Financial toxicity (FT), broadly defined as the objective financial burden and subjective financial distress associated with cancer and its treatment, is now recognized as a core component of high-quality cancer care. Contemporary evidence indicates that financial strain can contribute to treatment nonadherence, delayed or forgone care and deterioration in health-related quality of life (4, 5).

To characterize this patient-reported burden consistently, de Souza et al. developed the Comprehensive Score for Financial Toxicity (COST), a validated instrument that has since been widely used to assess FT among patients with cancer (6–12). Across cancer populations, lower COST scores, indicating more severe financial toxicity, have been consistently associated with poorer health-related quality of life, supporting the clinical relevance of FT as a patient-reported outcome. This issue may be particularly important in lung cancer because patients frequently undergo multimodal or prolonged treatment while experiencing substantial symptom burden, reduced work capacity, and ongoing supportive care needs (5, 13). Emerging lung cancer-specific evidence suggests that FT is both prevalent and persistent. For example, a prospective cohort study found that FT continued over time among patients with lung cancer, rather than being confined to a single phase of treatment (13). In metastatic non-small-cell lung cancer treated with immunotherapy or chemoimmunotherapy, substantial unmet supportive care needs and financial hardship have been reported (14). In China, a national survey found that 77.0% of patients with lung cancer experienced financial toxicity and 22.5% had moderate-to-severe toxicity (15). FT has also been documented in patients with resected lung cancer, indicating that the problem is not limited to advanced disease (16). Among lung cancer survivors, greater unmet needs were associated with lower quality of life and higher FT (17).

Despite this growing body of evidence, existing findings remain challenging to compare because studies have been conducted across diverse countries and health-care systems and have included different disease stages, treatment modalities, study populations, among other factors. A systematic synthesis of the available evidence is therefore needed to characterize the burden of FT among patients with lung cancer and its variation across different clinical and socioeconomic contexts. Accordingly, we conducted a systematic review and meta-analysis to estimate the prevalence and severity of FT and summarize its associated factors in this population.

## 2 Method

### 2.1 Study design and registration

This systematic review and meta-analysis evaluated FT among patients with lung cancer. Because this study was based on published data and did not involve individual-level patient information, institutional review board approval and informed consent were not required. This study followed the Preferred Reporting Items for Systematic Reviews and Meta-Analyses (PRISMA) reporting guideline and was registered in PROSPERO (CRD420251207694, 18).

### 2.2 Search strategy and eligibility criteria

A systematic literature search was conducted in November 2025 in PubMed, Embase, the Cochrane Library, and Scopus using terms related to lung cancer and financial toxicity. Studies were eligible if they were original investigations of patients with lung cancer written in English and reported financial toxicity, financial burden, financial hardship, or related patient-reported economic distress. Observational studies of any design were included if they provided data on the prevalence, severity, COST-Based scores (including COST and COST Patient-Reported Outcome Measure (COST-PROM) scale)^1^, correlates, or outcomes of FT. Studies that focused exclusively on health system costs rather than patient-level financial burden were excluded. Reviews, editorials, letters, conference abstracts without sufficient data, duplicate reports, and studies without extractable outcome data were also excluded. The complete search strategies for each database, see Supplementary Table S1.

### 2.3 Study selection and data extraction

After duplicate removal, 2 authors independently screened titles and abstracts and then assessed the full texts of potentially eligible articles. Disagreements were resolved by discussion, with adjudication by a third author when necessary.

Two authors independently extracted data using a standardized form. Extracted variables included first author, publication year, country, study design, sample size, patient characteristics, disease stage, treatment modality, FT measurement method, prevalence of FT, mean COST-Based score, and key associated factors or outcomes.

### 2.4 Quality assessment

Study quality was assessed independently by 2 authors using the National Institutes of Health Study Quality Assessment Tool for Observational Cohort and Cross-Sectional Studies. Discrepancies were resolved through discussion with a third author as needed. Each study was classified as good, fair, or poor quality on the basis of overall risk of bias (Supplementary Table S2).

### 2.5 Data synthesis and statistical analysis

#### 2.5.1 Qualitative synthesis

All eligible studies were included in the qualitative synthesis. Study characteristics, FT measurement approaches, definitions and thresholds, and principal findings were summarized descriptively. Factors associated with FT were categorized into sociodemographic, socioeconomic, clinical diagnosis and treatment, psychosocial, and health-system domains. Studies that assessed patient-level financial burden but did not provide data suitable for meta-analysis were included only in the qualitative synthesis.

A harvest plot was constructed to provide a structured visual synthesis of factors associated with FT. Each numbered bar represented one study, and its position indicated whether the reported factor was associated with greater or lower FT; horizontal position did not represent effect magnitude. Bar height represented study sample size, and bar color distinguished directly measured FT, objective financial burden, and contextual or moderating evidence. Evidence patterns were classified as “repeated” when supported by at least three unique studies and as “limited” when reported by one or two studies. When available, findings from adjusted analyses were prioritized. Because effect measures, reference categories, covariate adjustments, and statistical models varied substantially, the harvest plot was used for structured evidence mapping rather than quantitative effect estimation or formal certainty grading.

#### 2.5.2 Quantitative synthesis

Studies were included in the prevalence meta-analysis if the number of patients with FT and the total sample size were reported or could be derived from the published data. Studies were eligible for the continuous-outcome meta-analysis if they reported mean scores derived from COST-based instruments, including the COST and COST-PROM, together with sufficient measures of dispersion to estimate standard errors. For studies reporting medians with ranges or interquartile ranges, means and standard deviations were estimated using established approximation methods before quantitative synthesis. Random-effects meta-analyses were conducted to account for between-study variability. For prevalence outcomes, study-specific proportions were transformed using the logit transformation, pooled using a random-effects model, and subsequently back-transformed for presentation. For continuous outcomes, mean COST-based scores and their 95% confidence intervals (CIs) were pooled using inverse-variance random-effects models. Forest plots were used to present study-specific and pooled estimates.

#### 2.5.3 Subgroup analyses

Exploratory subgroup analyses were conducted, where sufficient data were available, according to country income level and disease stage (treatment method). Countries were classified according to the World Bank income classification (18). Disease stage (treatment method) was categorized as advanced disease or systemic treatment, mixed-stage or all-stage populations, and surgery-dominant populations. A further exploratory subgroup analysis was conducted based on the cutoff value used to define FT. Differences between subgroups were evaluated using a formal test for subgroup differences. Because several subgroups contained a limited number of studies, these analyses were considered exploratory and were interpreted cautiously.

#### 2.5.4 Sensitivity analyses

Leave-one-out sensitivity analyses were performed by sequentially excluding each study and recalculating the pooled estimate to evaluate whether the overall findings were disproportionately influenced by any individual study.

The feasibility of conducting a quality-based sensitivity analysis was also assessed. A restricted analysis excluding studies at higher risk of bias was planned if studies with different quality ratings were available.

#### 2.5.5 Heterogeneity

Statistical heterogeneity was evaluated using Cochran’s Q test, the I^2^ statistic, and the between-study variance (τ^2^). I^2^ values of less than 25, 25–75%, and greater than 75% were interpreted as indicating low, moderate, and high heterogeneity, respectively. For Cochran’s Q test, a two-sided *p* value of less than 0.10 was considered indicative of statistical heterogeneity. Where appropriate, 95% prediction intervals were calculated to estimate the range within which the underlying effect of a future comparable study might be expected to lie. Because prediction intervals may be imprecise when only a small number of studies are available, they were interpreted cautiously. For subgroup analyses, heterogeneity within each subgroup was assessed using Cochran’s Q, I^2^, and τ^2^, while differences between subgroup estimates were evaluated using a test for subgroup differences.

#### 2.5.6 Publication bias

Potential publication bias was explored through visual inspection of funnel plots and using Egger’s regression test and Begg’s rank correlation test. Because only eight studies were included in the prevalence meta-analysis and ten studies in the continuous COST-based score meta-analysis, these assessments had limited statistical power. Therefore, the results were considered exploratory and interpreted cautiously.

#### 2.5.7 Statistical analysis

Random-effects models were fitted using restricted maximum likelihood estimation, with Hartung–Knapp adjustments applied to the calculation of confidence intervals and subgroup tests. All statistical tests were two-sided, and *p* values of less than 0.05 were considered statistically significant, except for Cochran’s Q test, for which *p* < 0.10 was used. All statistical analyses were performed using R software 4.5.3.

## 3 Results

### 3.1 Study selection

**Figure 1:**
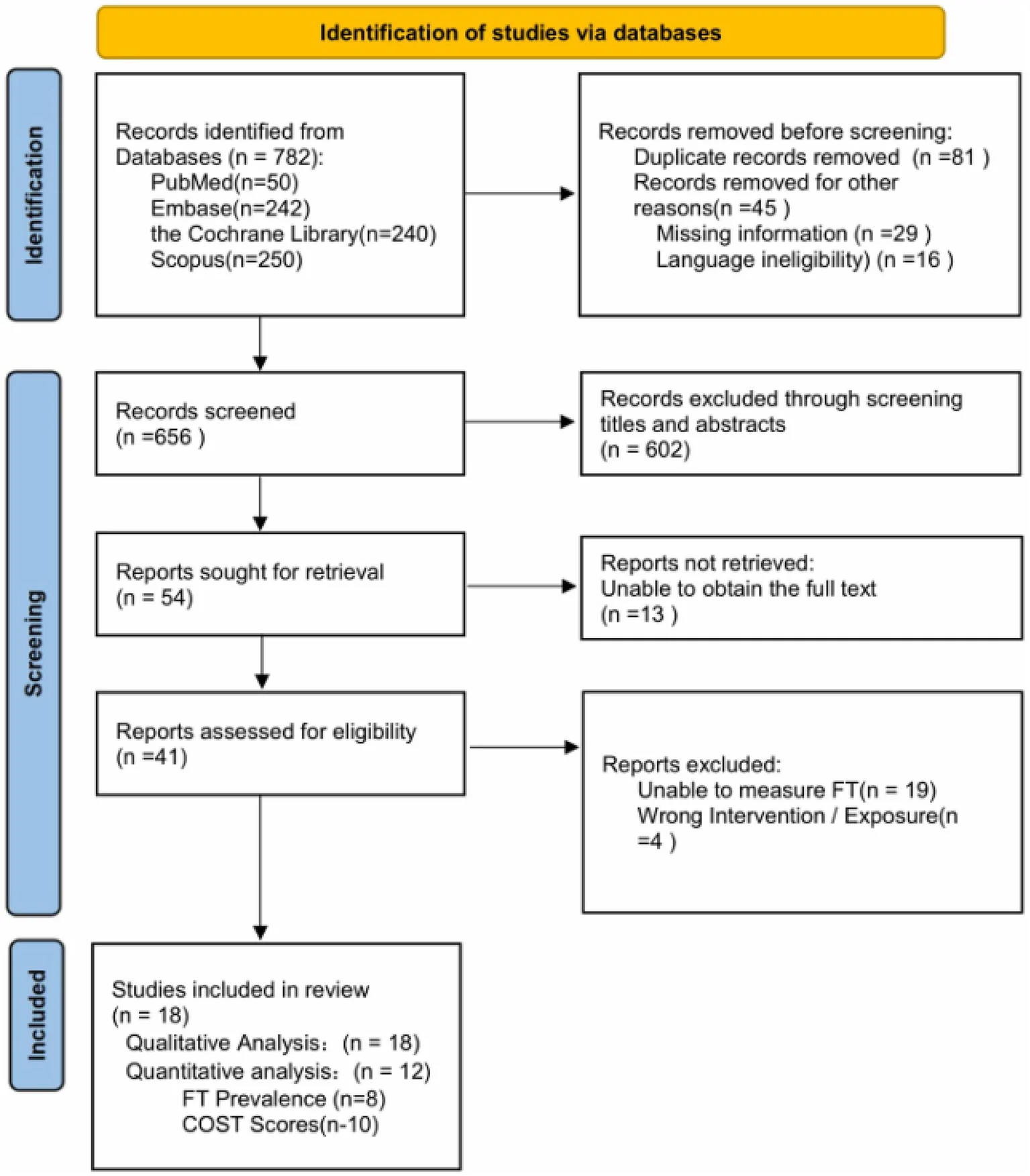
Flow diagram of included studies.

A total of 782 records were identified through database searching. After removal of 126 records before screening, 656 records remained for title and abstract screening, of which 602 were excluded. 54 reports were sought for retrieval, and 41 full-text articles were assessed for eligibility after 13 reports could not be retrieved. Following full-text review, 18 studies were included in the systematic review (7–10, 12–17, 19–26). Reasons for exclusion included inability to measure financial toxicity (*n* = 19), and wrong intervention or exposure (*n* = 4) (Figure 1).

### 3.2 Quality assessment

Across the 18 included studies, 4 were rated as good quality (20–23) and 14 as fair quality (14–17, 24–33); no study was rated as poor. The main limitations were the frequent absence of sample size justification or power calculation, limited ability to establish temporal sequence between exposure and outcome in cross-sectional designs, insufficient observation period for some prognostic questions, and incomplete reporting on assessor blinding or repeated exposure assessment. Overall, the included evidence was methodologically acceptable but predominantly observational and cross-sectional, indicating a moderate overall risk of bias (Supplementary Table S3).

### 3.3 Study characteristics

The 18 included studies were published between 2018 and 2025. Sample sizes ranged from 43 to 384,340 participants. Fourteen studies used a cross-sectional design, whereas 4 were cohort studies. Geographically, the included studies were conducted primarily in China (8 studies) (15, 24, 26, 27, 30–33) and the United States (7 studies) (14, 16, 17, 21–23, 28), with 1 study each from Japan (25), South Korea (20), and Canada (29). Most studies focused on specific clinical subgroups, including patients with advanced or metastatic lung cancer, postsurgical populations, survivors of lung cancer, and patients receiving immunotherapy (Table 1).

**Table 1:** Summary of study characteristics.

Source	Country	Study design	Sample size	Age (Mean/Median)	Primary treatment modality	Quality assessment
Xu et al. (31)	China	Cross-sectional	152	Median 62.1 years (range 32–84)	Continuous multimodality therapy (stage III–IV, non-surgical)	Fair
Hazell et al. (28)	US	Cross-sectional	131	Median 65 years (range 35–90)	First-line therapy (chemotherapy, radiation, or multimodality) for stage II–IV	Fair
Liu et al. (15)	China	Cross-sectional	843	Groups: <55 yrs. (30.5%), 55–65 yrs. (43.5%), >65 yrs. (26.0%)	Inpatient treatment (all stages, mixed modalities)	Fair
Cui et al. (32)	China	Cross-sectional	218	Mean 59.7 years (URRBMI: 60.7; UEBMI: 57.6)	Mixed treatments (chemotherapy, radiotherapy, targeted therapy, etc.) for ≥2 months	Fair
Xiao et al. (24)	China	Cross-sectional	447	Median 51 years (range 22–87)	Surgery (post-surgical patients)	Fair
Hsu et al. (17)	US	Cross-sectional	232	Median 69 years (IQR: 60.5–75.0)	Surgery (53.9%), radiation (58.2%), chemotherapy (59.1%), immunotherapy (34.9%), targeted therapy (41.8%)	Fair
Deboever et al. (16)	US	Cross-sectional	463	Mean 64.8 years	Surgery (lobectomy 62.8%, minimally invasive 46.7%), some with neoadjuvant therapy	Fair
Shan et al. (26)	China	Cross-sectional	218	Mean 49.9 ± 9.6 years	Chemotherapy history (47.4%), radiotherapy history (40.8%), mixed stages	Fair
Ito et al. (25)	Japan	Cross-sectional	147	Median 70 years (IQR: 63–75)	Chemotherapy (88.3%), chemoradiation (10.9%), best supportive care (0.7%) for stage IIIB–IV	Fair
Yoo et al. (20)	South Korea	Cohort	47	Median 62 years (range 41–81)	Nivolumab (standard dose 3 mg/kg Q2W or low dose 20/100 mg Q3W) for stage IIIB–IV NSCLC	Good
Jenkins et al. (21)	US	Cohort	138,219	Median 69 years (IQR: 62–75)	Surgical resection (lobectomy 72.1%, sublobar 26.5%, pneumonectomy 0.8%) for stage IA	Good
Zhang et al. (27)	China	Cross-sectional	421	Median 62 years (IQR: 52–69)	Mixed treatments (chemotherapy, radiotherapy, immunotherapy, targeted therapy, surgery) for all stages	Fair
Klein et al. (22)	US	Cohort	43	Mean 66 ± 10 years	Concurrent chemoradiotherapy (definitive) for locally advanced NSCLC	Good
Ng et al. (23)	US	Cohort	384,340	Median 68 years (insured); 59 years (uninsured)	Surgical resection (lobectomy, pneumonectomy, esophagectomy) for lung/esophageal cancer	Good
Ezeife et al. (29)	Canada	Cross-sectional	200	Median 65 years (range 32–86; mean 64.3 ± 10.9)	Systemic therapy (IV chemotherapy, oral TKI, immunotherapy), some on clinical trials	Fair
McLouth et al. (14)	US	Cross-sectional	60	Mean 62.5 ± 9.3 years (median 62.5, range 40–82)	Immunotherapy (pembrolizumab 75%, nivolumab 21.7%, atezolizumab 3.3%) with or without chemotherapy for stage IV NSCLC	Fair
Li et al. (30)	China	Cross-sectional	273	Groups: <40 yrs. (9.2%), 40–49 yrs. (13.6%), 50–59 yrs. (31.9%), 60–69 yrs. (28.2%), ≥70 yrs. (17.2%)	Mixed treatments (chemotherapy 86.8%, radiotherapy 64.8%, targeted therapy 39.6%, immunotherapy 50.9%, surgery 33.3%)	Fair
Zhang et al. (33)	China	Cross-sectional	322	Mean 62.36 years (range 34–74)	Immunotherapy (some combined with chemotherapy/targeted therapy)	Fair

### 3.4 FT definitions and measures

Considerable heterogeneity was observed in the definition and measurement of financial toxicity across studies. Of the 18 studies included, the vast majority (*n* = 13) used COST-based as a measure of financial toxicity, with only 5 studies using self-reporting economic burden or defined by household income/self-paying expenses ratio. Even in studies using the COST scale, the cut-off value selection for FT differs (e.g., <26, <24, <22, <21) (Table 2).

**Table 2:** Summary of FT measurement modalities and outcomes.

Source	FT definitions	Measurement of FT	Results
Xu et al. (31)	FT was rated as strong by a score larger than the cohort’s median COST	COST	The median value of the COST scores was 25.5 (range 4–42, mean ± SD, 9.7 ± 0.8)
Hazell et al. (28)	COST <24 as FT	COST	38.2% (*n* = 50) reported that they were either ‘just getting on’ or ‘struggling’ financially. 22.9% (*n* = 30) were unable to afford basic necessities over the past year
Liu et al. (15)	COST <26 as FT; 14–25 mild FT; 0–13 moderate/severe FT	COST	Altogether, 77% (*n* = 649) of the sample patients had FT (COST <26), 54.5% (*n* = 459) had mild FT (COST 14–25), and 22.5% (*n* = 190)had moderate and severe FT (COST 0–13). mean score of FT was 20.32 (SD 9.514), and the median score was 20 (interquartile range 14–25).”
Cui et al. (32)	with lower scores indicated higher FT	COST	In unmatched data: URRBMI patients had lower COST scores (mean 13.39) and higher MCCB (21.56%) vs. UEBMI (COST 19.51, MCCB 9.86%). URRBMI, COST, Mean (SD):13.39(8.73) In matched data: COST and MCCB differences became insignificant. Multivariate logit regression (matched): Higher FT (lower COST) was associated with higher MCCB (OR = 0.94, 95% CI: 0.89–0.98). Insurance moderated the relationship: For URRBMI patients, higher FT predicted higher MCCB (OR = 0.89, 95% CI: 0.83–0.95).
Xiao et al. (24)	a lower score indicating more severe FT.	COST-PROM	Nearly half (42.51%, n = 190) of the participants experienced financial toxicity. the COST-PROM median score of 22 (range: 1–44)
Hsu et al. (17)	lower scores reflect higher FT	COST plus unmet needs with quality of life	The incidence of FT in this study was not directly presented The median overall COST score was 28 (IQR, 22–34).
Deboever et al. (16)	COST<26 as FT	COST	There were 196 patients (42.3%) who experienced FT. The median FT score reported was 29 (interquartile range: 21–37)
Shan et al. (26)	COST <22 as FT	COST-PROM	There are 122 (56%) participants with FT.
Ito et al. (25)	NA	self-reported outcomes	A little financial problem 44(31.4%); Quite a bit 16(11.4%); Very much 6(4.3%)
Yoo et al. (20)	Lower economic status and higher drug costs	No direct FT scale; examined low-dose nivolumab cost as a cost-conscious treatment strategy	18 patients received low-dose nivolumab, faced high pressure of self-financing
Jenkins et al. (21)	NA	household income quartiles used as socioeconomic exposure	Lowest income group (Q1): 21,508 (15.6%). low-income patients exhibited worse overall survival, lower treatment quality, stacked risk factors
Zhang et al. (27)	lower scores indicate more severe FT	COST-PROM	Median FT score was 16 (IQR: 9–24); mild FT (19.47%), moderate FT-resource deficient (7.84%), moderate FT balanced (35.63%), and severe FT (37.06%)
Klein et al. (22)	NA	self-reported outcomes	12 (28%)answered‘a little’,1 (2%)answered‘quite a bit’and 6 (14%)answered ‘very much’.
Ng et al. (23)	FT risk defined as health expenditure exceeding 40% of postsubsistence income	compared hospitalization costs (for uninsured) or maximum out-of-pocket costs (for insured) with postsubsistence income	Approximately 68.9% (*n* = 2,648) of uninsured and 17.3% (*n* = 65,826) of insured patients were at risk of financial toxicity
Ezeife et al. (29)	COST <21 as FT	COST	Because 21 is the median score for the entire cohort, this means that exactly half of the patients had a score below 21 and half had a score above 21. 100 (50%) had financial problems, the median COST value reported was 21 (range, 0–44; mean± SD, 21.2 ± 10.1).
McLouth et al. (14)	higher score represents less financial distress.	COST and self-reported outcomes	63% (*n* = 38) reported psychological hardship, 57% (*n* = 34)reported behavioral hardship, and 33% (*n* = 20) reported material hardship.” “More than half (52%, *n* = 31) reported hardship in at least two of the three domains; 27% (*n* = 16) reported hardship in all three domains financial distress, the FACIT-COST score mean was 23.84, SD = 9.96
Li et al. (30)	COST <22 as FT	COST	the median COST score among patients with lung cancer was 19.00 (12.00, 25.00), with 65.9% (*n* = 180) of patients experiencing high FT.
Zhang et al. (33)	higher total scores suggesting a lower FT	COST	The mean COST score was 23.47 ± 6.98. FT and self-perceived burden both had direct effects on QoL; self-perceived burden partially mediated the association between FT and QoL, accounting for 33.9% of the total effect.

COST, Comprehensive Score for Financial Toxicity; PROM, Patient-Reported Outcome Measures.

### 3.5 Qualitative synthesis

**Figure 2:**
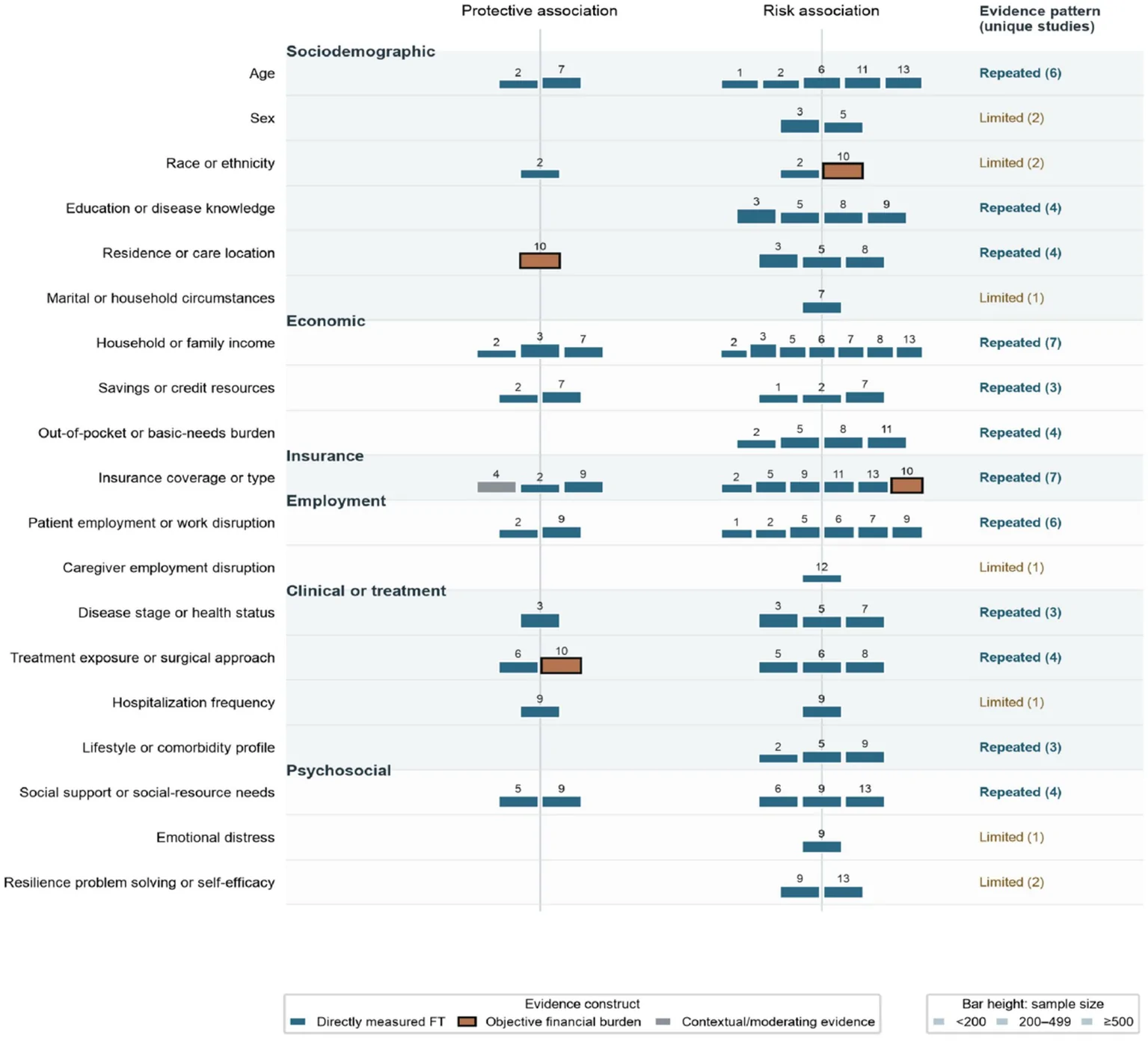
Harvest plot of risk and protective factors related to FT. Each numbered bar represents one study. Bar position indicates the reported direction of association rather than effect magnitude, bar height represents sample size, and bar color indicates the evidence construct. “Repeated” indicates evidence from at least three unique studies, whereas “limited” indicates evidence from one or two studies. The plot represents structured evidence mapping and should not be interpreted as a formal certainty-of-evidence assessment. 1 Xu et al. (31); 2 Hazell et al. (28); 3 Liu et al. (15); 4 Cui et al. (32); 5 Xiao et al. (24); 6 Hsu et al. (17); 7 Deboever et al. (16); 8 Shan et al. (26);9 Zhang et al. (27); 10 Ng et al. (23)*; 11 Ezeife et al. (29); 12 McLouth et al. (14); 13 Li et al. (30). *Good quality; all other studies were rated Fair. "Repeated" indicates ≥3 studies and "limited" 1-2 studies; this is structured evidence mapping, not a formal certainty rating.

Based on the 18 included studies, we systematically mapped the pathways from upstream structural determinants to downstream patient outcomes in the development of FT among lung cancer patients. Meanwhile, the relevant influencing factors are presented using a Harvest plot (Figure 2).

#### 3.5.1 Upstream structural influencing factors

Across the included studies, healthcare insurance systems differed substantially across countries/regions and showed a strong association with FT risk. Cui et al. (32) compared two types of health insurance in a Chinese cohort and found that patients covered by the Urban Rural Resident Basic Medical Insurance (URRBMI) had significantly lower COST scores than those with Urban Employee Basic Medical Insurance (UEBMI) (mean 13.39 vs. 19.51), indicating that less generous insurance coverage is directly linked to higher FT. Ng et al. (23), using a U. S. based sample, further demonstrated that 68.9% of uninsured patients were at risk of FT, compared with only 17.3% of insured patients – a nearly four fold difference. These findings underscore that both the scope and depth of health insurance coverage serve as key upstream structural determinants of FT. Additionally, Yoo et al. (20) observed in a Korean population that patients receiving low dose nivolumab faced heavy financial pressure due to full out of pocket payment, reflecting the direct impact of drug pricing and reimbursement policies on FT burden.

#### 3.5.2 Individual economic vulnerability

Individual level socioeconomic vulnerability constitutes an important intermediate step in the pathway to FT. Jenkins et al. (21) used household income quartiles as the exposure measure and reported that patients in the lowest income group (Q1, 15.6% of the cohort) not only had higher FT risk but also exhibited worse overall survival, lower treatment quality, and stacked risk factors. Ezeife et al. (29), using a cut off of COST-based < 21 (the cohort median) in a Canadian population, reported that 50% of patients experienced financial problems, with a median COST-based of 21 (range 0–44). Xu et al. (31) further identified that having less than one month of savings and being employed but on sick leave were significant risk factors for FT, highlighting that employment stability and financial reserves are core dimensions of individual economic vulnerability.

#### 3.5.3 Cancer treatment costs and income loss

The direct and indirect economic shocks generated during treatment constitute the driving force that transforms vulnerability into manifest FT. Hazell et al. (28) reported that 38.2% of patients described their financial situation as either “just getting on” or “struggling,” and 22.9% were unable to afford basic necessities in the past year, reflecting the erosion of daily living by treatment related expenditures. McLouth et al. (14) approached the issue from the caregiver perspective and found that caregivers reduced work hours or even quit their jobs to care for patients, further amplifying household income loss. Importantly, in that study, 63% of patients reported psychological hardship, 57% reported behavioral hardship, and 33% reported material hardship; more than half (52%) experienced hardship in at least two domains, and 27% in all three. This reveals the multifaceted financial distress arising from the combination of treatment costs and income reduction.

#### 3.5.4 Patient outcomes

The ultimate impact of FT manifests in patients’ treatment behavior and health outcomes. Zhang et al. (34), through mediation analysis, found that FT not only directly reduced quality of life (QoL) but also indirectly affected QoL via self perceived burden as a mediator, with the mediating effect accounting for 33.9% of the total effect-suggesting that FT amplifies its negative impact on patient outcomes through psychological pathways. Jenkins et al. (21) further observed that low income patients (Q1 group) had significantly worse overall survival and lower treatment quality, with risk factors showing a cumulative effect. Liu et al. (15) also indicated in their study that FT was significantly correlated with patients’ self rated health status and disease stage. Patients with advanced disease carried a heavier clinical burden and poorer subjective health perception, which were associated with a higher risk of FT. More importantly, the study investigated patient coping behaviors adopted in response to financial difficulties: approximately 38% of patients with moderate to severe FT had considered quitting treatment, and as FT severity increased, the proportions of patients reporting delayed treatment, poor medication adherence, and poor follow up visit adherence all showed a significant upward trend. These findings extend the conceptualisation of FT from a mere “economic burden” to a driver of “treatment behavior modification,” revealing that FT may be an important antecedent factor contributing to reduced treatment adherence among patients with lung cancer.

### 3.6 Quantitative synthesis

Among the included studies, eight studies provided directly poolable binary data on FT, and ten studies reported continuous COST-based scores suitable for meta-analysis. All studies contributing to either quantitative synthesis were cross-sectional and were rated as fair quality. The meta-analysis on the prevalence of FT showed that individual study estimates ranged from 0.42 to 0.77, and the overall proportion of FT was 0.55 (95% CI, 0.43–0.66) (Supplementary Figure S1). The meta-analysis of the COST-based scores showed that the average scores of each study ranged from 16.33 to 29.00, and the mean combined COST-base score was 22.91 (95% CI, 20.57–25.25) (Supplementary Figure S2).

### 3.7 Subgroup analyses

**Figure 3:**
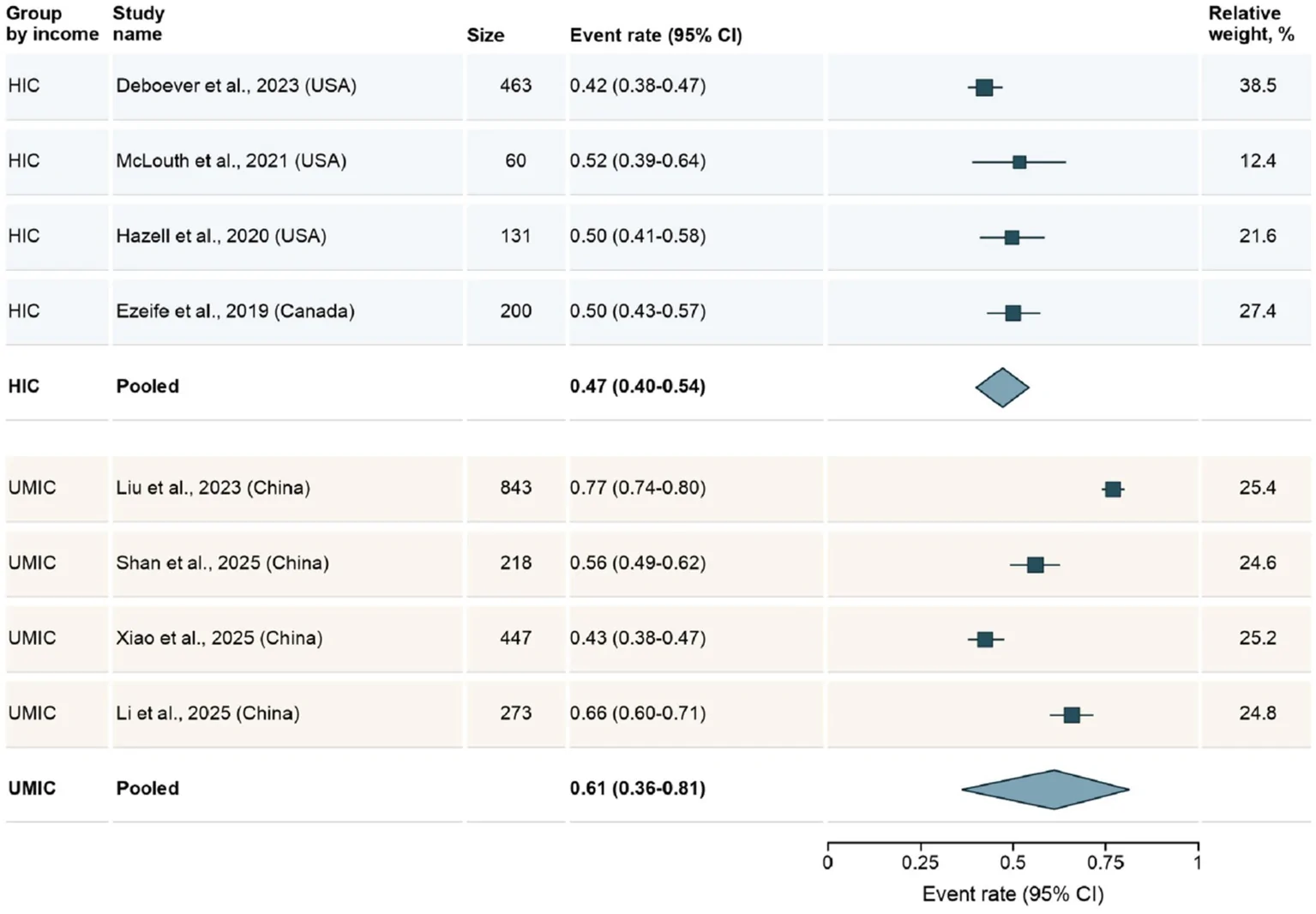
Forest plot for FT prevalence, subgrouped by HICs and UMICs. Random-effects model (REML) with Hartung-Knapp 95% Cls. Test for subgroup differences: *F* (1, 6) = 2.42, *p* = 0.171.

**Figure 4:**
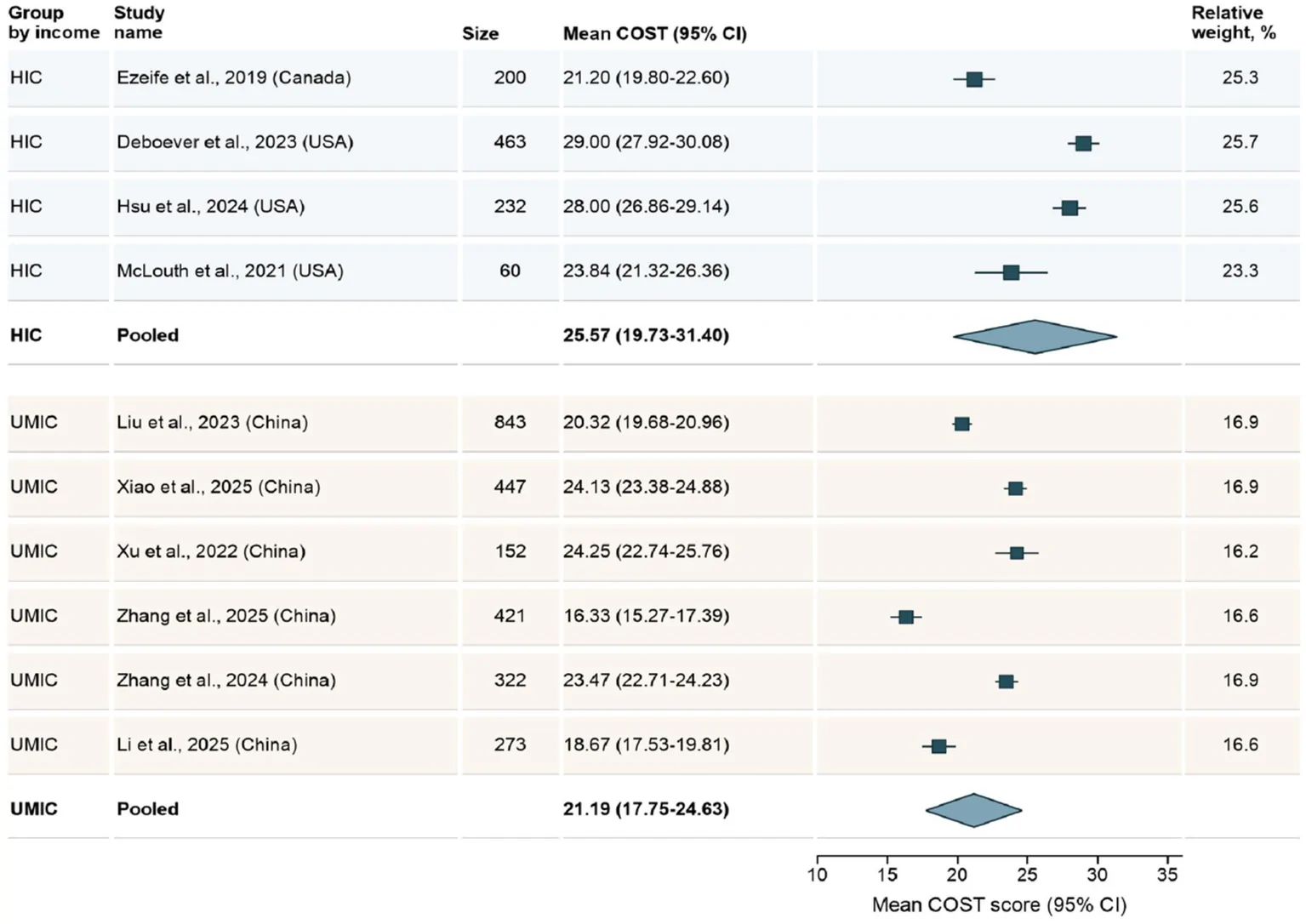
Forest plot for mean COST-based score, subgrouped by HICs and UMICs. Random-effects model (REML) with Hartung-Knapp 95% CIs. Test for subgroup differences: *F* (1, 8) = 3.89, *p* = 0.084.

When studies were grouped according to country income level, the pooled FT proportion was 0.47 (95% CI, 0.40–0.54) in high-income countries (HICs) and 0.61 (95% CI, 0.36–0.81) in upper-middle-income countries (UMICs). The difference between income groups was not statistically significant (*p* = 0.171) (Figure 3). For continuous COST-based scores, the pooled mean was 25.57 (95% CI, 19.73–31.40) in HICs and 21.19 (95% CI, 17.75–24.63) in UMICs. Although the lower mean score in UMICs directionally consistent with more severe FT, the subgroup difference did not reach statistical significance (*p* = 0.084) (Figure 4).

**Figure 5:**
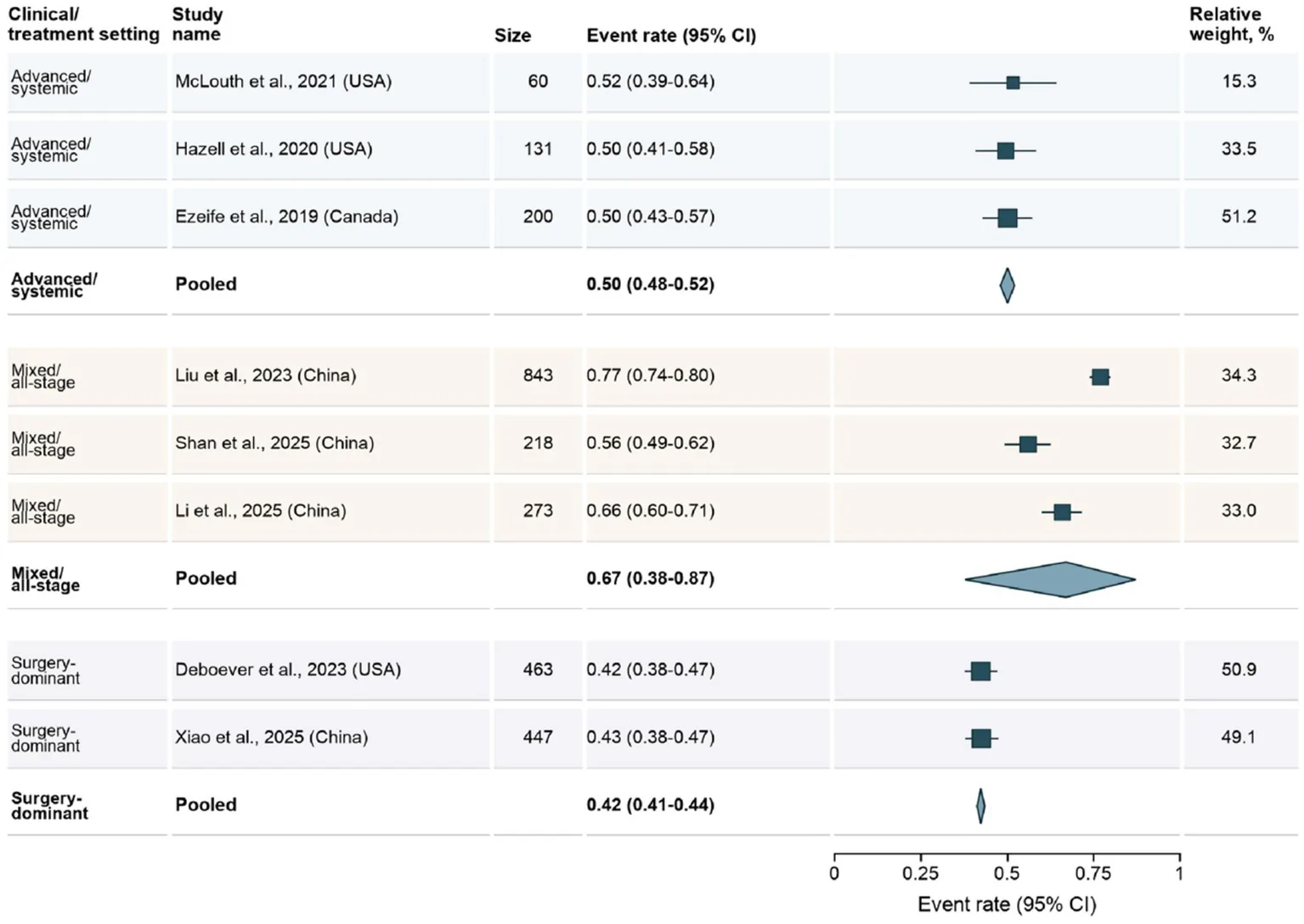
Forest plot for FT prevalence, subgrouped by disease stage (treatment method). Random-effects model (REML) with Hartung-Knapp 95% Cls. Test for subgroup differences: *F* (2, 5) = 7.48, *p* = 0.031.

**Figure 6:**
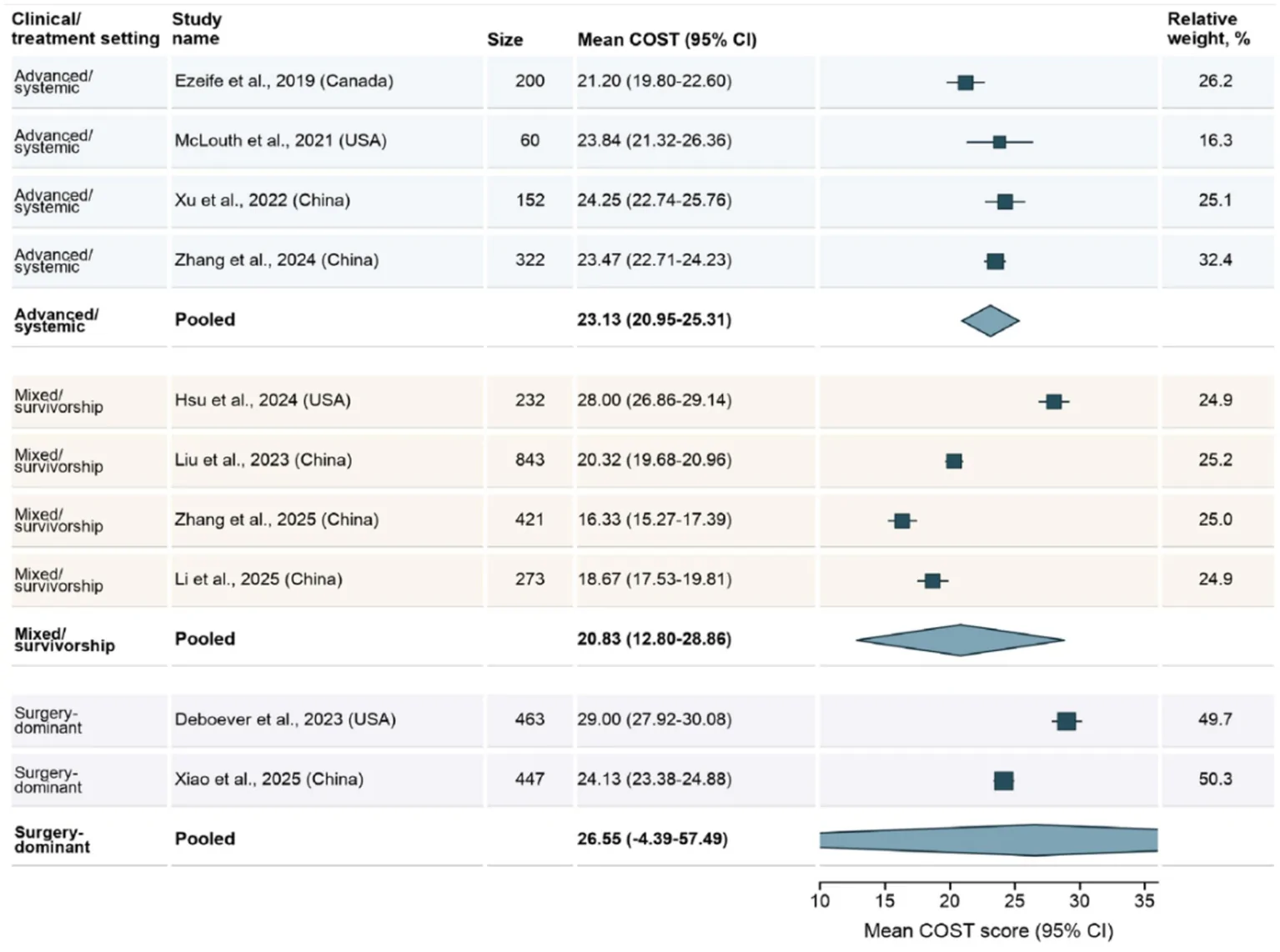
Forest plot for mean COST-based score, subgrouped by disease stage (treatment method). Random-effects model (REML) with Hartung-Knapp 95% Cls. Test for subgroup differences: *F* (2, 7) = 1.66, *p* = 0.258.

In the prevalence analysis, the pooled proportion was 0.50 (95% CI, 0.48–0.52) among studies of patients with advanced disease or receiving systemic treatment, 0.67 (95% CI, 0.38–0.87) among mixed-stage or all-stage populations, and 0.42 (95% CI, 0.41–0.44) among surgery-dominant populations. The test for subgroup differences was statistically significant (*p* = 0.031) (Figure 5). For COST-based scores, the pooled mean was 23.13 (95% CI, 20.95–25.31) in advanced-disease or systemic-treatment populations, 20.83 (95% CI, 12.80–28.86) in mixed-stage or survivorship populations, and 26.55 (95% CI, −4.39 to 57.49) in surgery-dominant populations. The subgroup difference was not statistically significant (*p* = 0.258) (Figure 6).

Among studies using COST-based thresholds to define FT, the pooled proportion was 0.58 (95% CI, 0.37–0.76) for cutoff values of 21–22 and 0.57 (95% CI, 0.15–0.91) for cutoff values of 23–25. No evidence of a subgroup difference was observed (*p* = 0.999) (Supplementary Figure S3).

### 3.8 Sensitivity analyses

Leave-one-out sensitivity analyses were performed by sequentially excluding each study and refitting the primary REML/Hartung–Knapp model. For FT prevalence, the recalculated pooled proportions ranged from 0.51 to 0.57 (Supplementary Figure S4). For continuous COST-based scores, the recalculated pooled means ranged from 22.22 to 23.65 (Supplementary Figure S5). No individual study materially changed the direction or magnitude of either pooled estimate.

### 3.9 Heterogeneity

For FT prevalence, residual heterogeneity was moderate in the HIC subgroup (I^2^ = 45.2%) but remained substantial in the UMIC subgroup (I^2^ = 97.3%). When stratified by disease stage (treatment method), no detectable heterogeneity was observed in the advanced/systemic-treatment and surgery-dominant subgroups (both I^2^ = 0%), whereas substantial heterogeneity remained in the mixed/all-stage subgroup (I^2^ = 94.6%). For mean COST-based scores, substantial residual heterogeneity persisted across most income and clinical-setting subgroups (I^2^ = 73.7–99.0%), indicating that these subgroup variables explained only part of the between-study heterogeneity (Supplementary Table S4).

### 3.10 Publication bias

Publication bias was explored by visual inspection of funnel plots and by Egger regression and Begg rank correlation tests for both the FT prevalence and the continuous COST score meta-analysis (Supplementary Figure S6, S7). For the prevalence analysis, neither Egger testing (*p* = 0.687) nor Begg testing (*p* = 1.00) indicated statistically significant funnel plot asymmetry. For the continuous COST score analysis, Egger testing (*p* = 0.734) and Begg testing (*p* = 0.773) were likewise not statistically significant. However, because only 8 studies were included in the prevalence analysis and the number of studies remained modest in the continuous COST analysis, these findings should still be interpreted cautiously.

## 4 Discussion

This systematic review and meta-analysis provides a comprehensive synthesis of FT among patients with lung cancer. Across eight studies, the pooled proportion of patients meeting study-specific definitions of financial toxicity was 0.55 (95% CI, 0.44–0.65), while the pooled mean COST-based score across ten studies was 22.91 (95% CI, 20.10–25.73). Exploratory subgroup analyses provided further context for these overall estimates. The pooled prevalence was numerically lower in high-income countries than in upper-middle-income countries (0.47 vs. 0.61), although the between-group difference was not statistically significant (*p* = 0.171). Similarly, the mean COST-based score was higher in high-income countries than in upper-middle-income countries (25.57 vs. 21.19), suggesting numerically less severe financial toxicity in high-income settings, but this difference did not reach statistical significance (*p* = 0.084). By disease stage (treatment method), the pooled prevalence was 0.50 among patients with advanced disease or receiving systemic treatment, 0.67 in mixed-stage or all-stage populations, and 0.42 in surgery-dominant populations, with a statistically significant subgroup difference (*p* = 0.031). In contrast, subgroup differences in continuous COST-based scores were not statistically significant (*p* = 0.258). Leave-one-out analyses showed that the pooled estimates changed only modestly after the exclusion of individual studies; however, the persistence of substantial heterogeneity and the broad prediction intervals indicate that the pooled values should be interpreted as exploratory averages across heterogeneous settings rather than as universally applicable estimates.

Several factors may explain the observed patterns across country-income and clinical subgroups. FT is shaped not only by household income but also by insurance coverage, reimbursement policies, out-of-pocket payments, employment protection, availability of social assistance, and the duration and intensity of cancer treatment. Previous reviews have shown that younger age, lower income, limited savings, unemployment, insufficient insurance protection, and greater out-of-pocket expenditure are consistently associated with more severe financial hardship among patients with cancer (35). Importantly, FT may remain prevalent even in countries with publicly funded or universal health-care systems because patients can still incur noncovered medication costs, transportation and accommodation expenses, loss of employment income, and informal caregiving costs (36). Conversely, patients in middle-income settings may face greater direct payments and less comprehensive social protection, although substantial variation also exists within each national income category (37, 38). Therefore, the absence of statistically significant income-group differences in the present study should not be interpreted as evidence that health-system and economic contexts are unimportant. Rather, it may reflect the small number of countries represented, substantial within-group heterogeneity, and limited statistical power for subgroup comparisons. Otherwise, measurement instruments may also perform differently across languages and cultures because perceptions of financial distress, willingness to disclose economic hardship, and expectations regarding family financial support vary internationally. Future cross-country studies could be considered therefore combine standardized patient-reported FT measures with context-specific information on out-of-pocket expenditure, household income, insurance coverage, employment effects, and relevant health policies.

The clinical subgroup findings should likewise be interpreted cautiously. Patients with advanced lung cancer or those receiving systemic therapy may experience prolonged treatment, repeated imaging and monitoring, medication-related expenditure, travel requirements, and sustained work disruption. Patients undergoing surgery may have a more time-limited treatment course, but they may still experience substantial short-term expenses, postoperative work loss, rehabilitation needs, and caregiver burden. The apparently higher prevalence in mixed-stage populations may partly reflect differences in study composition, assessment timing, financial-toxicity thresholds, and health-care settings rather than disease stage alone. In a prospective lung cancer cohort, Friedes et al. found that financial toxicity changed considerably at the individual level during follow-up and that its predictors could vary over the disease trajectory, suggesting that a single assessment may not fully characterize patients’ cumulative financial experiences (13). Consequently, although the significant prevalence subgroup test suggests that clinical and treatment setting may contribute to between-study variability, these observational subgroup results should not be interpreted as demonstrating that early diagnosis or surgery independently prevents financial toxicity.

The substantial heterogeneity observed in both quantitative analyses reinforces the importance of this distinction. For the prevalence outcome, I^2^ was 94.8%, and the 95% prediction interval ranged from 0.25 to 0.82. For continuous COST-based scores, I^2^ was 98.3%, and the prediction interval ranged from 13.66 to 32.16. These wide intervals indicate that the underlying FT burden in a future comparable study could differ considerably from the pooled average. Several overlapping factors may explain this variability, including different FT definitions and thresholds, variation in disease stage and treatment intensity, active treatment versus survivorship settings, differences in patient age and employment status, and variation in household financial resources. Cultural adaptation and interpretation of COST-based items may introduce additional differences across populations. Health-system factors, including insurance generosity, reimbursement arrangements, drug prices, out-of-pocket requirements, employment protection, and social welfare provision, are also likely to influence patient-level financial experiences.

The present findings have several implications for the prevention and management of financial toxicity in lung cancer care. First, financial distress might be assessed prospectively and repeatedly rather than only after severe hardship has developed. Screening could be undertaken at diagnosis, before major treatment decisions, during changes in systemic therapy, following surgery or hospitalization, and during survivorship follow-up. A validated COST-based instrument may be combined with brief questions about out-of-pocket expenses, income loss, transportation needs, insurance problems, and difficulty paying for basic necessities. The European Society for Medical Oncology consensus recommendations similarly support systematic identification and management of financial toxicity throughout the cancer-care trajectory (4). Second, screening could be linked to a defined response pathway. Patients with lower income, limited savings, unstable employment, inadequate insurance protection, substantial travel requirements, or intensive and prolonged treatment may benefit from early referral to financial navigators, social workers, insurance counselors, or patient-assistance programs. Navigation can include clarification of insurance benefits, applications for medication assistance and disability benefits, transportation and accommodation support, debt-management referral, and coordination of employment or caregiver resources. Although pilot studies have reported improvements in COST scores after comprehensive financial-navigation programs, evidence from controlled studies remains mixed, and a recent meta-analysis did not demonstrate a consistent overall improvement across all patients (7, 39). These findings suggest that navigation may be most effective when targeted to patients with substantial baseline financial risk and when accompanied by concrete financial assistance rather than information alone. Third, clinicians could incorporate cost communication into shared decision-making when clinically equivalent treatment options are available. Discussions may address anticipated medication costs, monitoring requirements, travel burden, treatment duration, employment consequences, and available financial assistance, while ensuring that patients are not directed toward clinically inferior care solely because of affordability. Existing evidence indicates that patients are generally willing to discuss treatment costs, but such discussions are often limited by insufficient information, time constraints, and uncertainty regarding professional responsibility (34, 40). Training clinicians and embedding financial counselors within multidisciplinary lung cancer teams may help translate screening results into timely intervention.

## 5 Limitations

Several additional limitations should be acknowledged. First, FT definitions, measurement instruments, and positive thresholds were inconsistent, and the prevalence synthesis combined participants meeting different study-specific definitions rather than a single diagnostic standard. Second, COST-based scores were obtained from different countries, languages, cultural contexts, and clinical populations. Some studies reported means directly, whereas others required conversion from medians and ranges or interquartile ranges, introducing additional uncertainty. Although analyses according to summary-statistic source did not identify a statistically significant difference, the number of studies was limited. Third, most included studies were cross-sectional, preventing causal or temporal interpretation of the identified factors and consequences. Fourth, adjusted effect estimates could not be quantitatively pooled because the studies used different effect measures, reference categories, covariate sets, and statistical models. Fifth, the evidence was geographically concentrated and did not represent low-income countries or the full diversity of global health-care systems. Sixth, subgroup analyses were exploratory, study-level, and based on small, sometimes overlapping categories, leaving considerable potential for ecological bias and residual confounding. Seventh, potential publication bias and small-study effects cannot be excluded. Although funnel plots and Egger and Begg tests did not indicate statistically significant asymmetry, these methods have limited power when fewer than 10 studies are available. Finally, the very high heterogeneity, wide prediction intervals, and limited ability to evaluate publication bias restrict the transportability of the pooled estimates.

## 6 Conclusion

In conclusion, financial toxicity represents an important but highly context-dependent patient-reported burden among patients with lung cancer. Worse FT is associated with socioeconomic disadvantage, inadequate insurance protection, employment disruption, clinical burden, and psychosocial distress. However, the pooled prevalence and COST-based score varied substantially across clinical, socioeconomic, measurement, and health-system contexts and were derived predominantly from fair-quality cross-sectional studies; they should therefore be interpreted as exploratory summaries rather than universally applicable benchmarks. Future studies should adopt standardized longitudinal assessments with transparent threshold reporting and prospectively evaluate risk-stratified, multilevel interventions linking early screening with financial navigation and structural financial protection.

## Data Availability

The original contributions presented in the study are included in the article/Supplementary material, further inquiries can be directed to the corresponding authors.

## Footnotes

1 The COST and COST-PROM were classified as COST-based instruments because they share the same 11 scored items, scoring range of 0–44, and score direction, with lower scores indicating more severe FT. The additional item in the COST-PROM is not included in the total score.
