# Supplementary material for: Financial toxicity among patients with lung cancer: a systematic review and meta-analysis
Source: Front Public Health. 2026 Aug 13;14:1896259. doi: 10.3389/fpubh.2026.1896259 (PMC13518355; doi:10.3389/fpubh.2026.1896259)

Supplementary Material

Table S 1.Complete Search Strategies for Each Database

Database 1	Pubmed
Date of search	11.10.2025
Language	Only English
Search terms	((financial toxicity[Title/Abstract]) OR (economic burden[Title/Abstract])) AND (lung cancer[Title/Abstract])
Database 2	Embase
Date of search	11.10.2025
Language	Only English
Search terms	Search:1. ('lung cancer'/exp OR 'lung tumor'/exp OR 'non small cell lung cancer'/exp OR 'small cell lung cancer'/exp)<br>2. ('financial toxicity'/exp OR 'financial hardship'/exp OR 'financial burden'/exp OR 'financial distress'/exp)<br>3. 1 AND 2
Database 3	the Cochrane Library
Date of search	11.10.2025
Language	Only English
Search terms	#1 MeSH descriptor: [Lung Neoplasms] explode all trees<br>#2 lung tumor* OR non small cell lung cancer* OR small cell lung cancer*<br>#3 #1 OR #2<br>#4 MeSH descriptor: [Health Expenditures] explode all trees<br>#5 financial toxicity* OR financial hardship* OR financial burden* OR financial distress*<br>#6 #4 OR #5<br>#7 #3 AND #6
Database 4	Scopus
Date of search	11.10.2025
Language	Only English
Search terms	(TITLE-ABS-KEY (Financial Toxicity) AND TITLE-ABS-KEY (lung cancer))
Reasons for excluding gray literature	In this systematic review, we did not include grey literature (e.g., conference abstracts, preprints, dissertations, clinical trial registrations without full results, or governmental/unpublished reports). This decision was made for three primary reasons. First, grey literature generally lacks formal peer review, which may compromise the methodological rigour and reliability of reported estimates, particularly for prevalence and association data. Second, such sources often provide insufficient detail on quantitative outcomes—such as odds ratios, regression coefficients, or subgroup-specific prevalence—necessary for our planned meta-analyses. Third, to avoid potential double-counting of overlapping patient cohorts, we restricted inclusion to definitively published versions of studies, as grey reports frequently precede later full-text journal publications.

Table S 2.Quality Assessment Tool for Observational Cohort and Cross-Sectional Studies.

Criteria	Yes	No	Other (CD, NR, NA)*
1. Was the research question or objective in this paper clearly stated?			
2. Was the study population clearly specified and defined?			
3. Was the participation rate of eligible persons at least 50%?			
4. Were all the subjects selected or recruited from the same or similar populations (including the same time period)? Were inclusion and exclusion criteria for being in the study prespecified and applied uniformly to all participants?			
5. Was a sample size justification, power description, or variance and effect estimates provided?			
6. For the analyses in this paper, were the exposure(s) of interest measured prior to the outcome(s) being measured?			
7. Was the timeframe sufficient so that one could reasonably expect to see an association between exposure and outcome if it existed?			
8. For exposures that can vary in amount or level, did the study examine different levels of the exposure as related to the outcome (e.g., categories of exposure, or exposure measured as continuous variable)?			
9. Were the exposure measures (independent variables) clearly defined, valid, reliable, and implemented consistently across all study participants?			
10. Was the exposure(s) assessed more than once over time?			
11. Were the outcome measures (dependent variables) clearly defined, valid, reliable, and implemented consistently across all study participants?			
12. Were the outcome assessors blinded to the exposure status of participants?			
13. Was loss to follow-up after baseline 20% or less?			
14. Were key potential confounding variables measured and adjusted statistically for their impact on the relationship between exposure(s) and outcome(s)?			

Quality Rating (Good, Fair, or Poor);*CD, cannot determine; NA, not applicable; NR, not reported.

Table S 3.NIH Quality Assessment Summary of Included Studies.

Source	Item1	Item2	Item3	Item4	Item5	Item6	Item7	Item8	Item9	Item10	Item11	Item12	Item13	Item14	Quality Rating
Xu2022	Yes	Yes	Yes	No	CD	No	No	Yes	Yes	No	Yes	CD	NA	Yes	Fair
Hazell2020	Yes	Yes	Yes	No	CD	No	No	Yes	Yes	No	Yes	CD	NA	Yes	Fair
Liu2023	Yes	Yes	No	Yes	CD	No	No	Yes	Yes	No	Yes	CD	NA	Yes	Fair
Cui2024	Yes	Yes	Yes	No	CD	No	No	Yes	Yes	No	Yes	CD	NA	Yes	Fair
Xiao2025	Yes	Yes	Yes	No	CD	No	No	Yes	Yes	No	Yes	CD	NA	Yes	Fair
Hsu2024	Yes	Yes	Yes	No	CD	No	No	Yes	Yes	No	Yes	NR	NA	Yes	Fair
Deboever2023	Yes	Yes	No	Yes	CD	No	No	Yes	Yes	No	Yes	NR	NA	Yes	Fair
Shan2025	Yes	Yes	Yes	No	CD	No	No	Yes	Yes	No	Yes	NR	NA	Yes	Fair
Ito2025	Yes	Yes	Yes	Yes	CD	No	No	Yes	Yes	No	Yes	NR	NA	Yes	Fair
Yoo2018	Yes	Yes	Yes	Yes	No	Yes	Yes	Yes	Yes	No	Yes	NR	NA	Yes	Good
Jenkins2025	Yes	Yes	Yes	Yes	No	Yes	Yes	Yes	Yes	No	Yes	NR	Yes	Yes	Good
Zhang2025	Yes	Yes	Yes	No	Yes	No	No	Yes	Yes	No	Yes	CD	NA	Yes	Fair
Klein2019	Yes	Yes	Yes	No	No	Yes	Yes	Yes	Yes	No	Yes	CD	Yes	Yes	Good
Ng2025	Yes	Yes	Yes	Yes	No	Yes	No	Yes	Yes	No	Yes	CD	NA	Yes	Good
Ezeife2019	Yes	Yes	Yes	No	CD	No	No	Yes	Yes	No	Yes	CD	NA	Yes	Fair
McLouth2021	Yes	Yes	Yes	Yes	No	CD	No	Yes	Yes	No	Yes	CD	Yes	No	Fair
Li2025	Yes	Yes	CD	Yes	No	CD	NA	Yes	Yes	No	Yes	CD	Yes	Yes	Fair
Zhang2024	Yes	Yes	Yes	Yes	Yes	No	NA	Yes	Yes	NA	Yes	CD	NA	No	Fair

Table S 4.Heterogeneity analysis of each subgroup.

subgroups	k	95% CI	Cochran Q（df）	P	I^2	τ^2
FT Prevalence						
HICs	4	0.47（0.40–0.54）	5.25（3）	0.155	45.2%	0.019
UMICs	4	0.61（0.36–0.81）	149.75（3）	<0.001	97.3%	0.405
advanced/systemic-treatment	3	0.50（0.48–0.52）	0.07（2）	0.965	0%	0
mixed/all-stage	3	0.67（0.38–0.87）	41.01（2）	<0.001	94.6%	0.225
surgery-dominant	2	0.42（0.41–0.44）	0.003（1）	0.958	0%	0
Mean COST-based Score						
HICs	4	25.57（19.73–31.40）	86.53（3）	<0.001	96.3%	12.94
UMICs	6	21.19（17.75–24.63）	213.56（5）	<0.001	98.1%	10.44
advanced/systemic-treatment	4	23.13（20.95–25.31）	10.43（3）	0.015	73.7%	1.36
mixed/all-stage	4	20.83（12.80–28.86）	235.78（3）	<0.001	99.0%	25.15
surgery-dominant	2	26.55（−4.39–57.49）	52.72（1）	<0.001	98.1%	11.63
Cutoff value used to define FT						
21–22	3	0.58（0.37–0.76）	12.57	0.002	83.8%	0.094
23–25	3	0.57（0.15–0.91）	157.45	<0.001	98.2%	0.644

Figure S 1. Forest Plot of FT Prevalence.

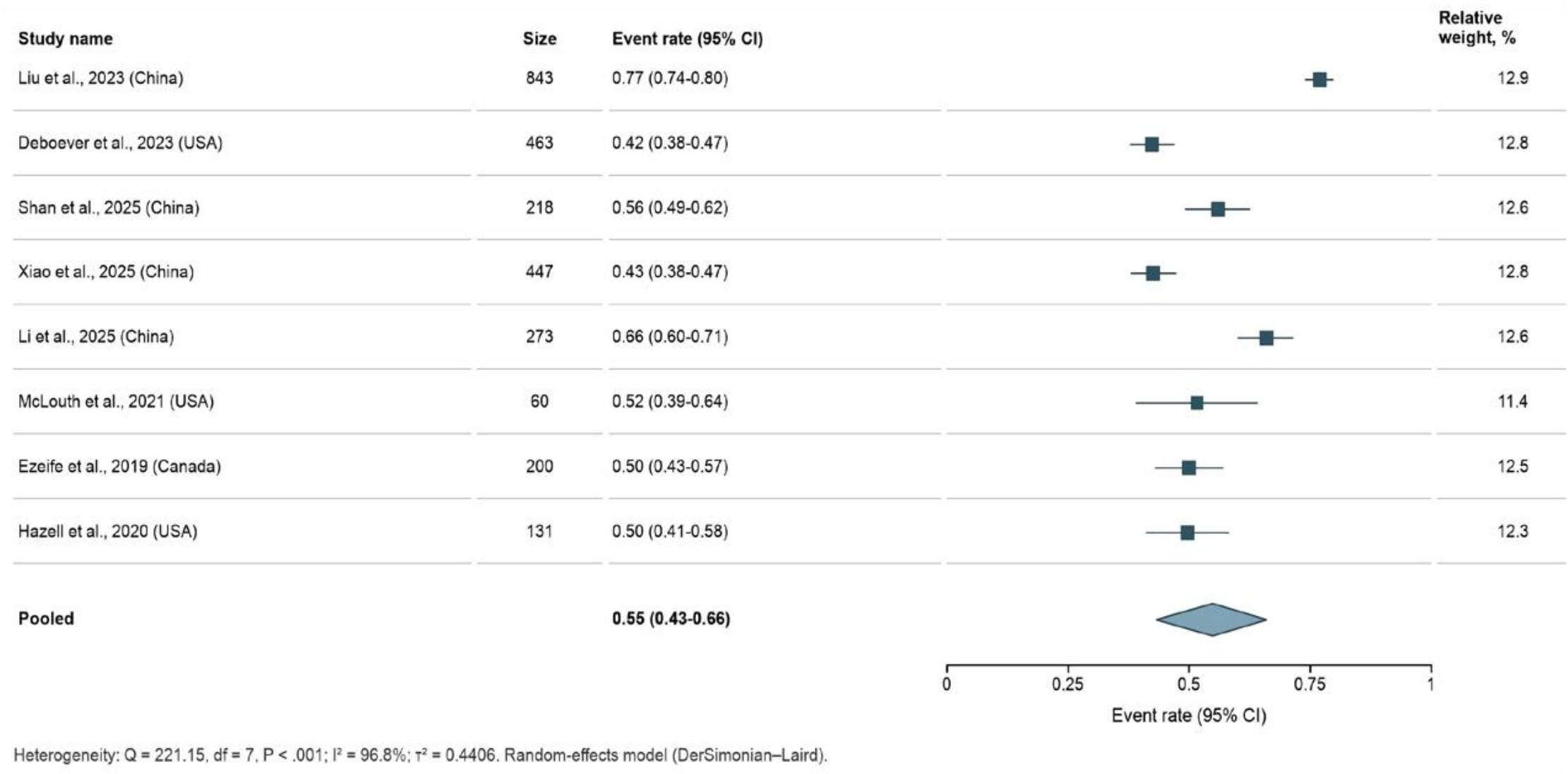

Figure S 2. Forest Plot of COST-based scores.

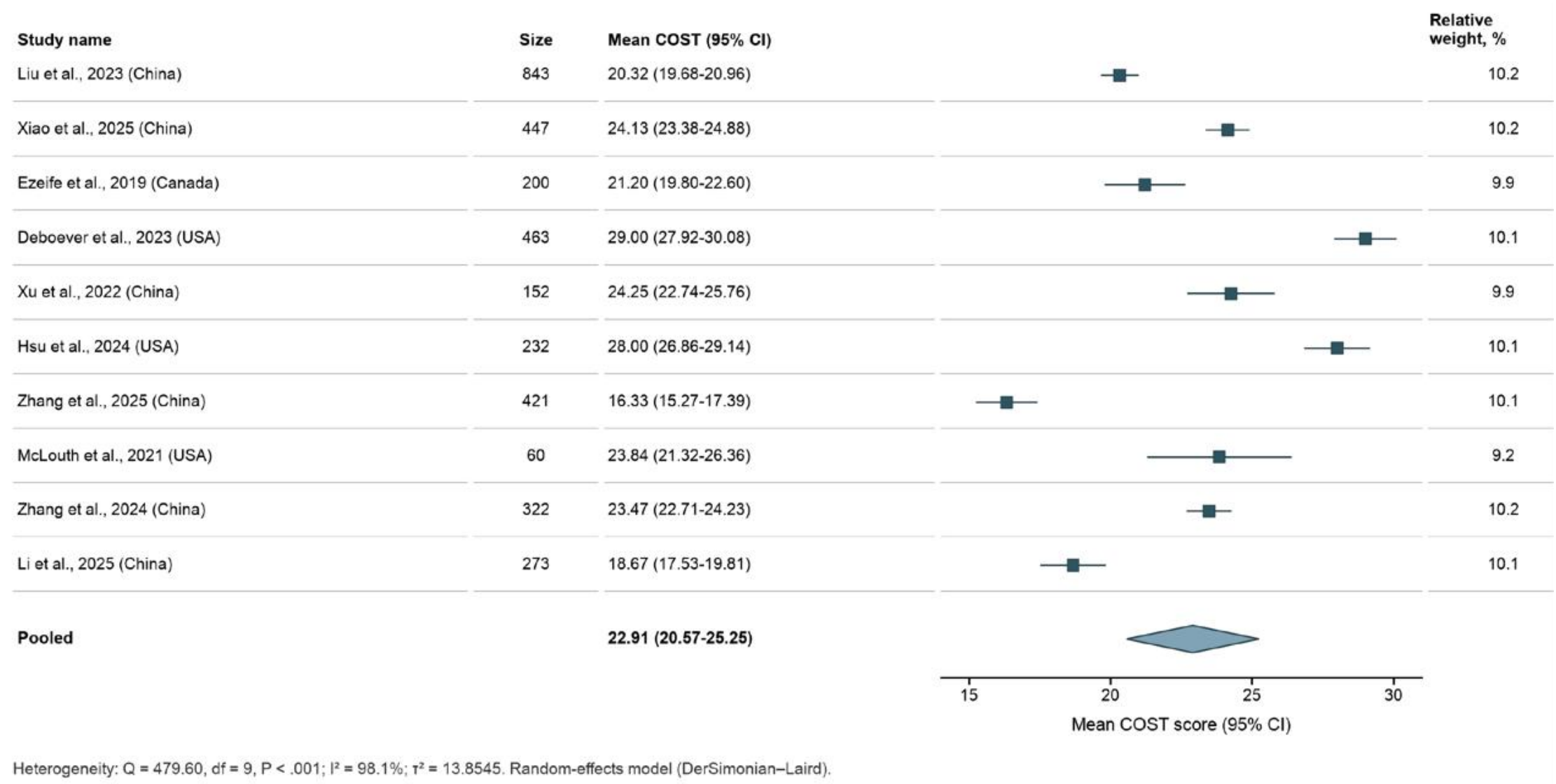

Figure S 3. Forest Plot for FT Prevalence, Subgrouped by COST Cut off Range.

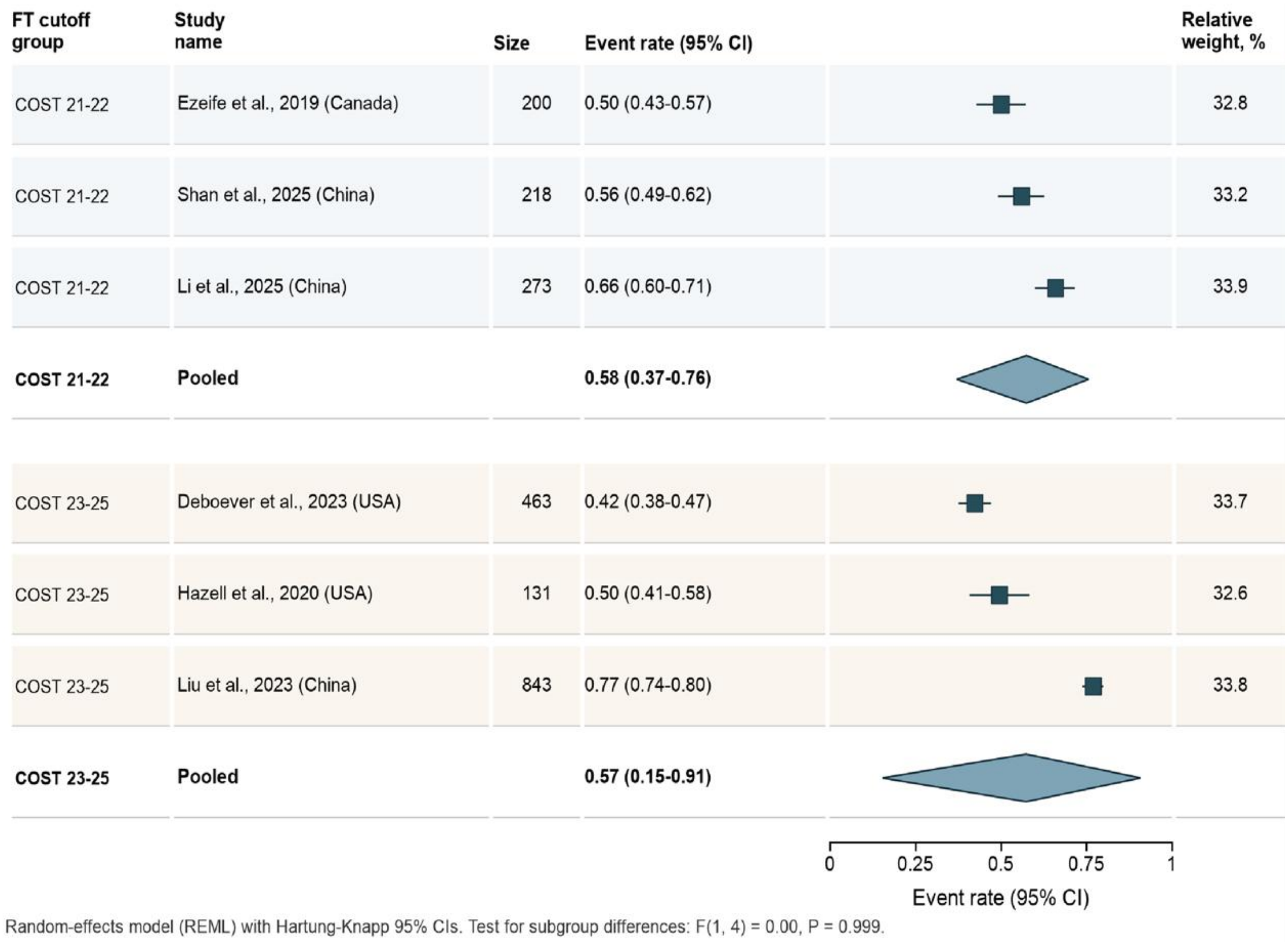

Figure S 4. Leave-one-out sensitivity analysis of FT prevalence.

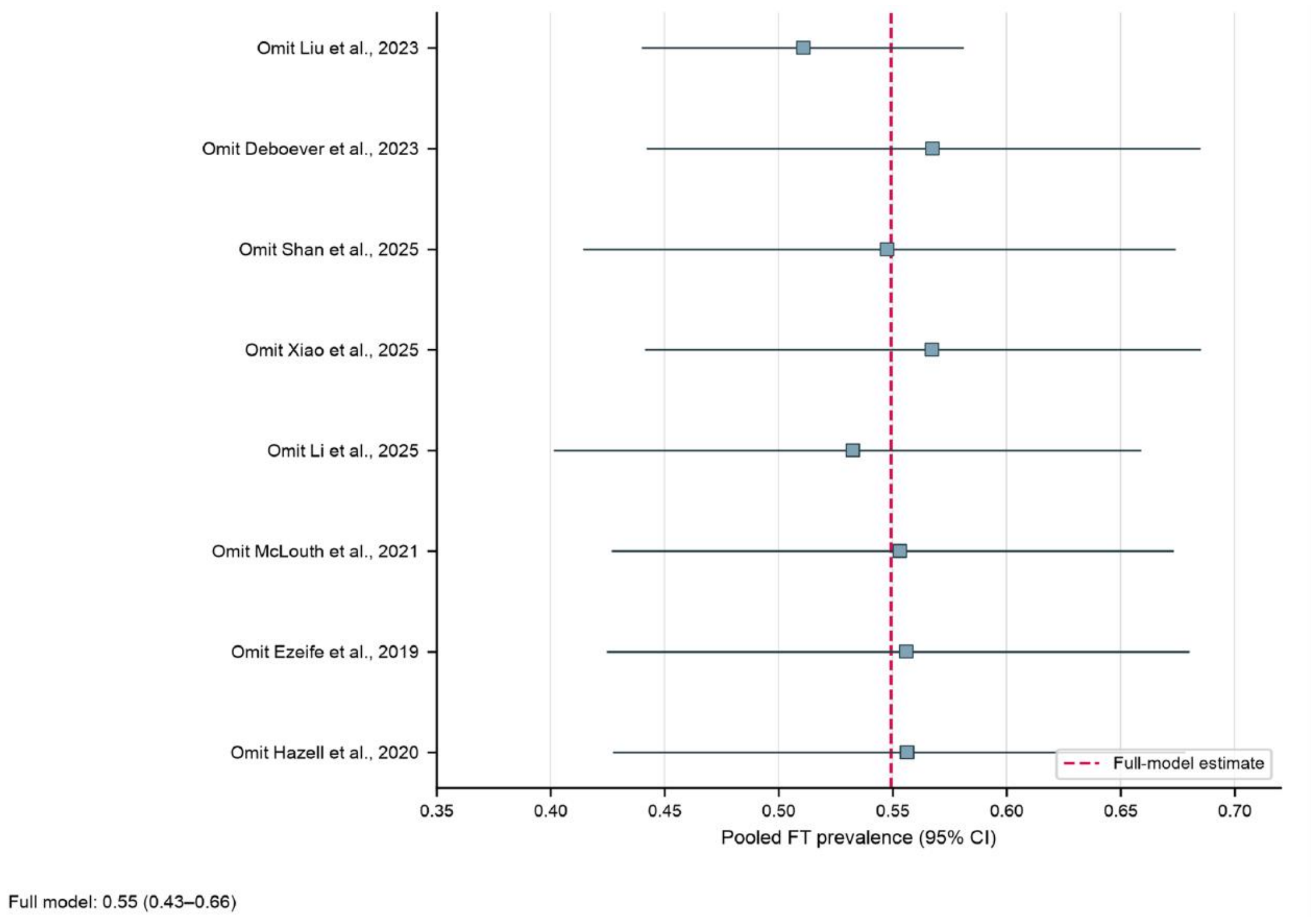

Figure S 5. Leave-one-out sensitivity analysis of COST-based Scores.

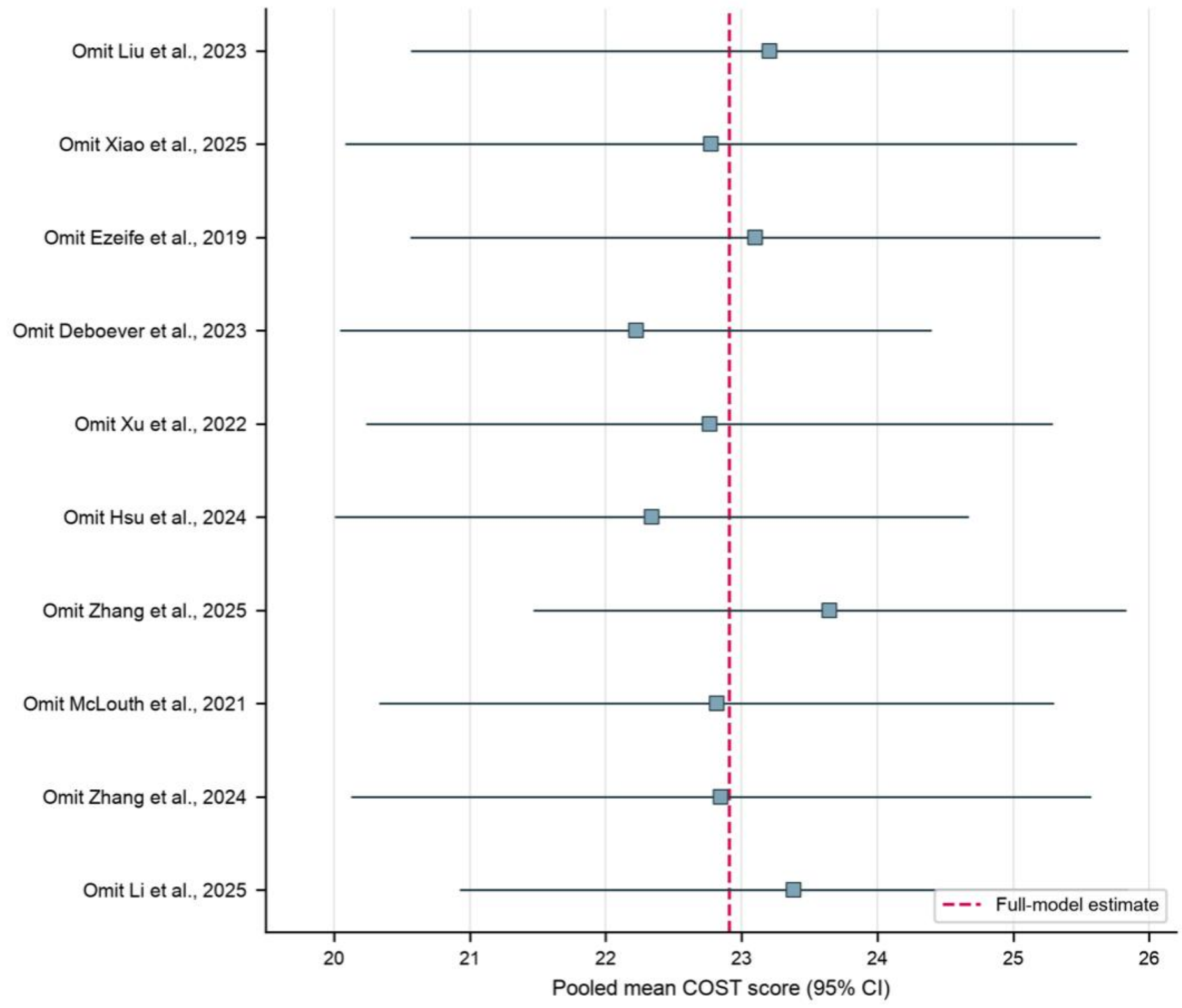

Full model: 22.91 (20.57–25.25)

Figure S 6. Funnel Plot of FT Prevalence.

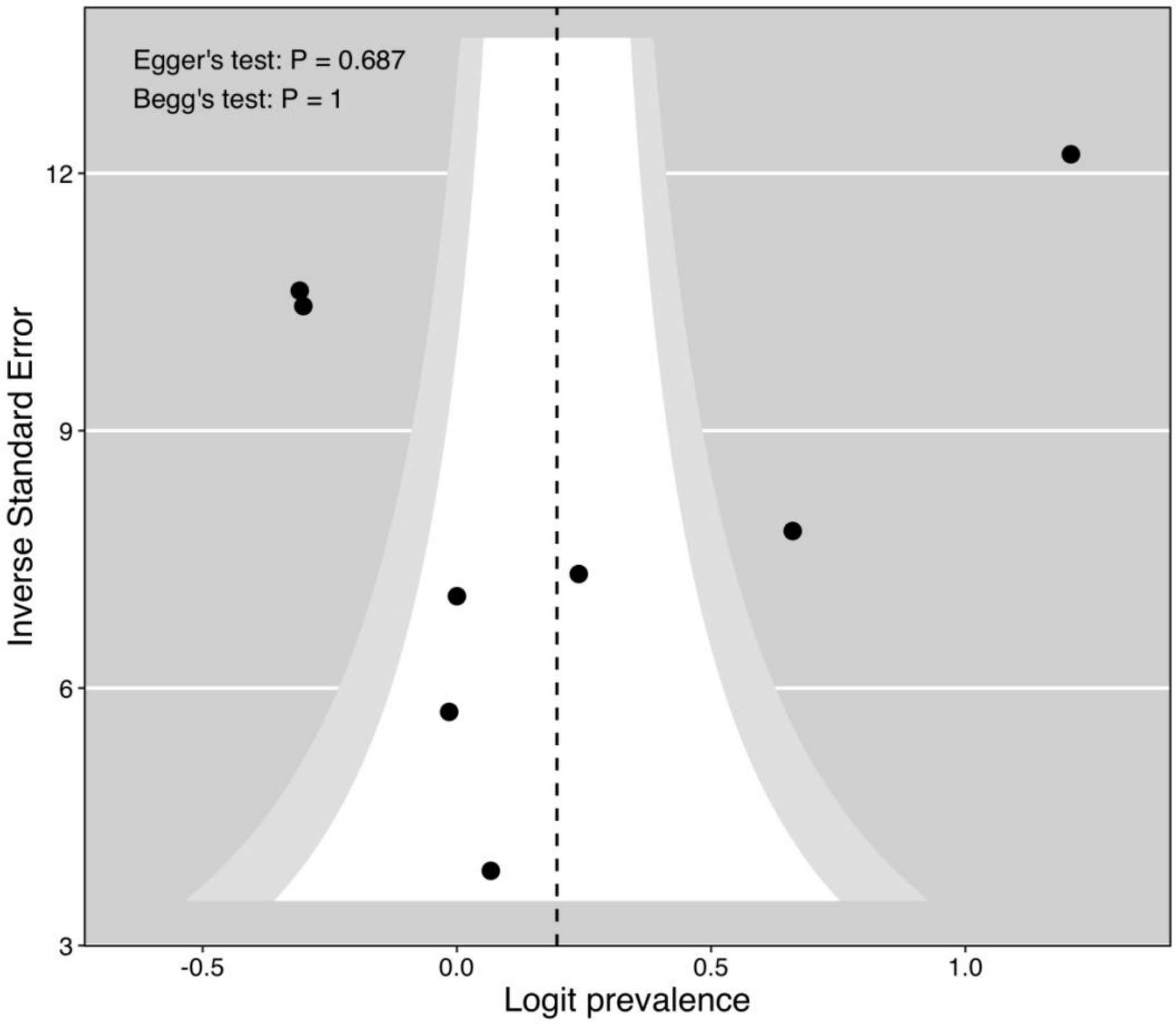

Figure S 7. Funnel Plot of COST-based Scores.

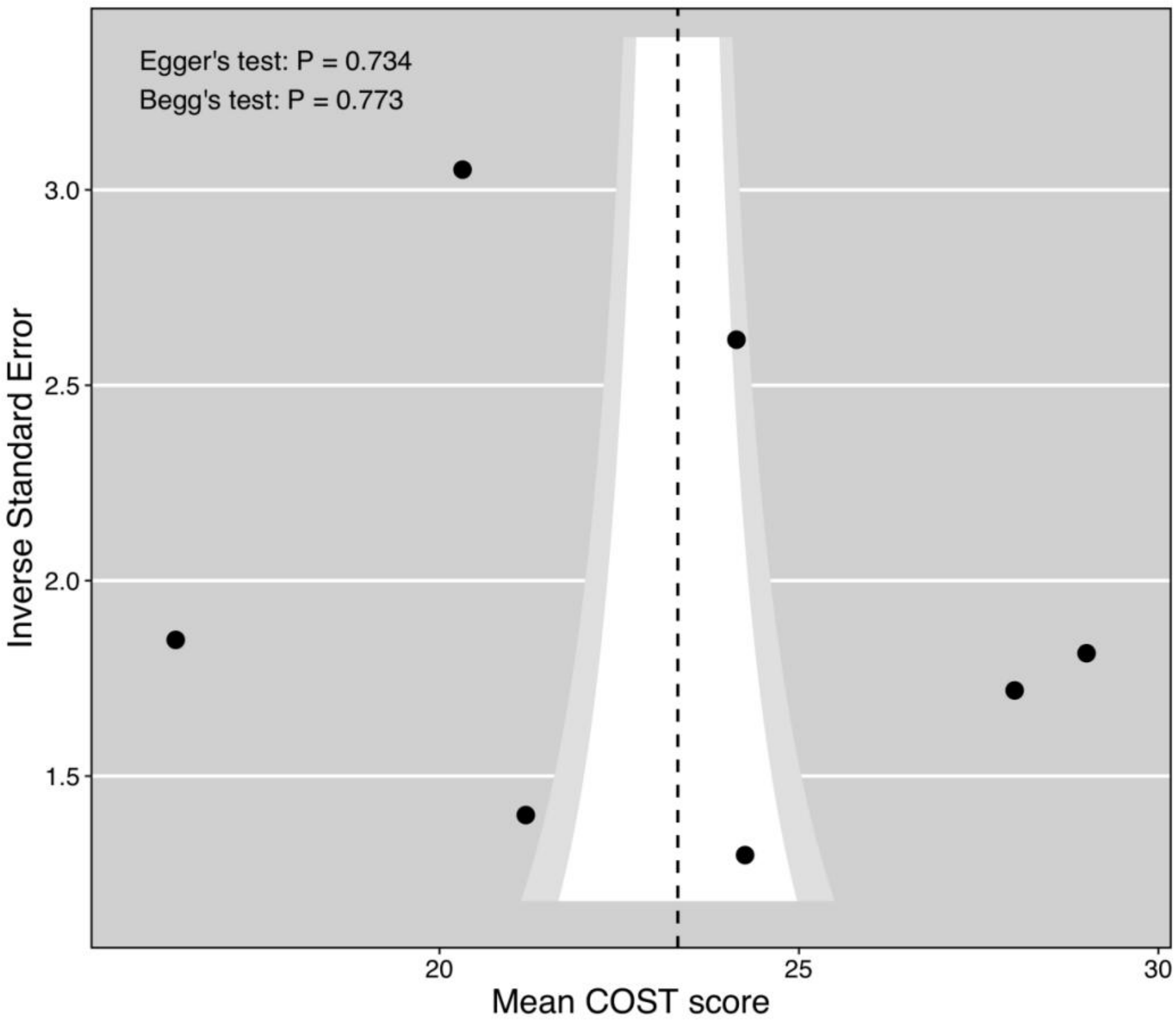

Supplement: Supplementary file 1 [file Supplementary_file_1.pdf]
